# Individual and family predictors of ultra-processed food consumption in Spanish children: The SENDO project

**DOI:** 10.1017/S136898002200132X

**Published:** 2022-05-27

**Authors:** Lorena García-Blanco, Víctor de la O Pascual, Arantxa Berasaluce, Laura Moreno-Galarraga, Miguel Ángel Martínez-González, Nerea Martín-Calvo

**Affiliations:** 1 San Juan Primary Care Health Center, Servicio Navarro de Salud-Osasunbidea, IdiSNA, Instituto de Investigación Sanitaria de Navarra, C/Irunlarrea 1, Pamplona 31080, Spain; 2 University of Navarra, Department of Preventive Medicine and Public Health, School of Medicine, IdiSNA, Instituto de Investigación Sanitaria de Navarra, Pamplona, Spain; 3 Department of Pediatrics, Complejo Hospitalario de Navarra B, IdiSNA, Instituto de Investigación Sanitaria de Navarra, Pamplona, Spain; 4 Biomedical Research Centre Network on Obesity and Nutrition (CIBERobn), Physiopathology of Obesity and Nutrition, Institute of Health, Carlos III. Av. Monforte de Lemos, Madrid, Spain

**Keywords:** Ultra-processed foods, Dietary attitudes, Eating habits, Nutritional knowledge, Screen time

## Abstract

**Background:**

Ultra-processed food (UPF) consumption is increasing exponentially, becoming a matter of concern for Public Health, given its adverse health effects.

**Objective:**

To identify individual and faGmily factors predicting UPF consumption in childhood.

**Design:**

The SENDO project is an ongoing prospective dynamic cohort of Spanish children. In this study, we used baseline information of participants recruited between January 2015 and June 2021. Dietary information was collected with a validated semi-quantitative FFQ, and food items were classified using the NOVA classification. Individual and family factors associated with UPF consumption (*P* < 0·20) in univariate analyses were introduced in a model of generalised estimating equations which accounted for intra-cluster correlations between siblings.

**Setting:**

The SENDO project (Spain), 2015–2021.

**Participants:**

Spanish children are recruited at the age of 4–5 years and followed yearly through online questionnaires completed by parents.

**Results:**

In this sample of 806 participants (49 % girls; mean age 5 years (sd: 0·90)), the mean UPF consumption was 37·64 % of total energy intake (sd: 9·59). Large family size and longer exposure to screens predicted higher consumption of UPF. On the other hand, better knowledge of children’s dietary recommendations, healthy dietary attitudes towards child’s eating habits and longer breastfeeding were associated with lower consumption of UPF. All these factors accounted for approximately 16 % of the variability on the consumption of UPF in childhood.

**Conclusion:**

Since most of the factors identified in this study are modifiable, they should be considered in public health strategies aimed at promoting healthy dietary habits in early life.

The NOVA food classification system categorises foods into four groups depending on the extent and purpose of processing. The first group includes unprocessed or minimally processed foods: none or mostly physical processing is used to make single whole foods more durable, accessible, convenient, palatable or safe. The second group contains processed culinary ingredients, which are substances obtained from the first group or from nature and may contain additives to preserve the original properties. The third group comprises processed foods made with the addition of substances such as salt, sugar or oil and processing techniques such as smoking, curing or fermentation without necessarily being an industrial processing. The fourth group includes ultra-processed foods (UPF) and beverages: industrial formulations made by adding sugar, oils, fats, salt, synthetic antioxidants and stabilisers to foods in group 1, which represent only a small proportion of the final product. They are ready-to-consume or ready-to-heat and thus require little or no culinary preparation, which makes them easily accessible and convenient but preserves a low nutritional quality. Typically, they are combined with sophisticated additives to make them durable and hyper-palatable^(1,2)^.

Over the last 20 years, probably as a consequence of industrialisation and globalisation^(3)^, the consumption of UPF has increased drastically worldwide, reaching the alarming proportion of 50 %–60 % of daily energy intake in several high-income countries^(4–7)^. Spain is not an exception since, in the last decade, the consumption of UPF increased from 11·0 % to 31·7 %^(8)^. While the evidence on the harmful effect of UPF consumption in children is still building, several studies in adult populations pointed out that UPF consumption is linked to a higher risk of obesity^(9)^, hypertension^(10)^ and all-cause mortality^(11,12)^.

Non-communicable diseases (i.e. CVD, diabetes, obesity and cancer) are the leading causes of mortality and morbidity in developed countries. The main risk factors related to those diseases are high blood pressure, excess weight, excessive energy intake and a poor quality diet (i.e. a diet poor in fruits and vegetables and rich in saturated fats). Nutrition plays an important role in the control of these factors, especially in childhood, since most eating habits are established in the first years of life and are maintained over time^(13)^. During infancy, children learn what, when and how much to eat based on the transmission of beliefs, attitudes and practices^(14)^ and are therefore, influenced by innate, familial and environmental factors^(15)^.

In a scenario where UPF consumption continues increasing and its harmful effects on children’s health have been already sufficiently reported^(16)^, it is essential from a public health perspective to identify modifiable factors associated with the consumption of UPF in early childhood. In this study, we aimed to identify individual and family factors that independently predict higher consumption of UPF in a sample of Spanish preschoolers.

## Material and methods

### Study population

The SENDO project (‘Seguimiento del Niño para un Desarrollo Óptimo’) is an ongoing prospective pediatric cohort focussed on studying the effect of diet and lifestyle on children’s and adolescents’ health. Participants are invited to enter the cohort by their pediatrician at their primary care health center or by the research team at school. The recruitment is permanently open. Inclusion criteria are: (1) age between 4 and 5 years; and (2) residence in Spain. The only exclusion criterion is the lack of access to an internet-connected device to complete the questionnaires. Information is collected at baseline and updated every year through online self-administered questionnaires completed by parents. For the present study, we used the baseline information of participants recruited between January 2015 and June 2021.

Of the 989 participants recruited before June 2021, 183 had not completed the baseline questionnaire at the time this study was started, and consequently were excluded. Therefore, the final sample for this study included 806 children with complete information.

The SENDO project follows the rules of the Declaration of Helsinki on the ethical principles for medical research in human beings. This study was approved by the Ethics Committee Committee for Clinical Research of Navarra (Pyto 2016/122). An informed consent was obtained from all participants’ parents at recruitment.

### Ascertainment of the outcome

Dietary information was collected through a previously validated semi-quantitative FFQ which included 149 food items^(17)^. For each food item, a portion size was specified. Parents reported how often their child had consumed each of the food items over the previous year by choosing one out of nine frequencies of consumption ranging from ‘never or almost never’ to ‘6 or more times/d’.

We considered free sugars as those added to foods and beverages by the industrial processing or in homemade preparations as well as those naturally present in honey, syrups and fruit juices^(18)^ and saturated fats are those fats in which the fatty acid chains have all single bonds. Nutrient content of each food item (including free sugars and saturated fats) was calculated by a team of dietitians by multiplying the frequency of consumption by the edible portion and the nutrient composition of the specified portion size based on the information of updated Spanish food composition tables and online databases^(19)^. Total energy intake (TEI) was obtained by adding the calorie contribution of each food item.

All the food items in the FFQ were classified by the extent and purpose of processing according to the NOVA classification (see online Supplemental Table 1). Some food items in the FFQ (croquet, ‘churro’, custard, lasagne, mayonnaise, muffin, pie, pizza and popcorn) could be classified in the NOVA 4 group (if they were industrialised products) or in the NOVA 3 groups (if they were homemade). Considering the dietary habits of Spanish families, and the results of the correlation of these food items with the rest of the NOVA groups (*r* with NOVA1 = -0·33, *r* with NOVA 2 = -0·11, *r* with NOVA 3 = -0·22 and *r* with NOVA 4 = 0·20), we think that participant’s UPF consumption was better captured when these items were included in the NOVA 4 group.

The percentage of TEI for each NOVA group was calculated by dividing the energy content of each group by TEI and multiplying the result by 100.

### Assessment of covariates

The participants’ parents completed a self-administered online questionnaire on sociodemographic, lifestyle and dietary habits at baseline. The age of the participants and their parents was calculated as the difference between the day the questionnaire was completed and their respective birthday.

BMI was calculated as the ratio between the reported weight (kg) and height-squared (m^2^). Nutritional status was defined using sex and age-specific cut-off points from the International Obesity Task Force^(20)^. Age- and sex-specific *Z*-score of the BMI was calculated using the LMS method^(20)^.

Physical activity was collected with a questionnaire that included fourteen activities classified as moderate or vigorous depending on the expected energy expenditure (below or over 5 METS/h). Participants indicated the time average dedicated to each activity during the previous year choosing from a list of ten possible responses for daily frequency and a list of four possible responses for the number of months/year. We calculated the time average (h/d) spent in moderate to vigorous physical activities within the previous year. Screen time was calculated as the mean time (h/d) spent watching television, using a computer or playing videogames during weekdays and weekends. Parental attitudes towards child’s dietary habits were evaluated through 8 yes/no questions (see online Supplemental Table 2). Affirmative answers were assigned 1 point and negative answers, 0 points. Hence, the final score ranged from 0 to 8 points, with a higher score suggesting healthier attitudes. Participants’ parents were classified as having unhealthy (0–3 points), average (4–6 points) or healthy attitudes (7–8 points) towards their child’s dietary habits. Unhealthy attitudes were used as the reference category. Parental nutritional knowledge was evaluated with questions on children’s dietary intake recommendations for eighteen different food groups (see online Supplemental Table 3). Each question was assigned 1 point if the answer met the dietary recommendations and 0 points if it did not^(21)^. The final score was expressed as a percentage, with higher value meaning higher knowledge on nutritional recommendations for children. Participants were categorised as having high (> 70 %), average (40–70 %) or low (<40 %) nutritional knowledge. Low knowledge was used as the reference category.

Other covariates were: participant’s sex (male or female), breastfeeding history (no breastfeeding, <6 months, 6–12 months, >12 months), maternal high education (yes or no), paternal high education (yes or no), number of children (1–2 children, 3–4 children, 5 or more), family history of obesity (yes or no), race (white or no), gestational age (< 38 weeks, 38–40 weeks, >40 weeks), birth weight (< 2500 g, 2500–3000 g, 3000–3500 g, 3500–4000 g, > 4000 g), participant’s position among siblings (the oldest/singleton, 2nd/3rd or 2nd–3rd/4, the youngest or beyond the 4th).

### Statistical analysis

We described participants’ characteristics by tertiles of UPF consumption. For descriptive purposes, we used percentages for categorical variables and means (SD) for quantitative variables. Linear trend tests across tertiles of UPF consumption were calculated by assigning the median of UPF consumption to each tertile and treating this variable as continuous in regression models.

In order to identify those factors that independently predict UPF consumption, the variables with a *P*-value below 0·20 in univariate analyses were introduced in a forward stepwise linear regression model with a *P* value for removal of 0·05. That model resulted in the adjusted differences (and 95 % CI) in the percentage of TEI (% kcal/d) (To convert energy to kJ, multiply kcal values by 4·184 kJ) associated with each predictor. Potentially causal associations were studied according to prior knowledge about biological plausibility and the existing evidence. We finally used generalised estimation equations models to calculate the magnitude of the association of each predictor after accounting for the intra-cluster correlation between siblings.

In further analyses, we calculated the multivariable-adjusted OR and 95 % CI for consuming more than 10 % of TEI as: (1) saturated fats; and (2) free sugars associated with each of the factors identified as independent predictors of UPF consumption in the main analysis^(19)^. In these analyses, generalised estimation equation models were also used to account for intra-cluster correlation between siblings.

Analyses were carried out using Stata version 15.0 (Stata Corp.) and all *P*-values are two-tailed.

## Results

This study included 806 participants (49 % girls) aged 5 years (sd: 0·90) who presented a mean UPF consumption of 37·64 % of TEI (sd: 9·59). The main characteristics of participants and their families by tertiles of UPF consumption are presented in Table 1. Although the questionnaire could be completed by either the father or the mother of the participant, we observed that mothers were the most frequent respondents (98 %).

**Table 1:** Characteristics of participants in the SENDO project and their families by tertiles of ultra-processed food consumption. Numbers are mean (sd) or *n* (%)

Columna1	T1		T2		T3		*P* for trend
	*n*	%	*n*	%	*n*	%	
*n*	269		269		268		
Range of the percentage of total energy intake	10·46–33·62		33·62–41·30		41·31–71·12		
Family’s characteristics							

	Mean	SD	Mean	SD	Mean	SD	
Paternal age (years)	41·04	5·21	40·39	5·67	40·97	5·25	0·85
Maternal age (years)	39·65	3·91	40	4·01	40·42	3·99	0·02

	*n*	%	*n*	%	*n*	%	
Maternal age							0·02
<35 years	36	13·38	25	9·29	22	8·21	
35–40 years	107	39·77	116	43·12	100	37·31	
40–45 years	103	38·29	100	37·17	114	42·53	
> 45 years	23	8·55	28	10·4	32	11·94	
Maternal high education	217	80·66	228	84·75	204	76·11	0·2
Paternal high education	163	60·59	170	63·19	152	56·71	0·37
Number of children							<0·001
1–2 children	199	73·97	154	57·24	146	54·47	
3–4 children	54	20·07	60	22·3	65	24·25	
5 or more	16	5·94	55	20·44	57	21·26	
Family history of obesity	56	20·97	50	18·93	51	19·17	0·59
Parental attitudes towards child’s dietary habits							<0·001
Unhealthy (0–3 points)	7	2·6	17	6·32	22	8·2	
Average (4–5 points)	64	23·76	91	33·82	110	41·04	
Healthy (6–8 points)	198	73·6	161	59·85	136	50·74	
Parental knowledge about child’s nutritional recommendations							<0·001
Low score (<40 %)	31	11·52	65	24·16	87	32·46	
Medium score (40–70 %)	183	68·03	168	62·45	167	62·31	
High score (>70 %)	55	20·44	36	13·38	14	5·22	
Children’s characteristics							
Sex (female)	147	54·64	123	45·72	127	47·38	0·08
Age (years)	4·83	0·74	5·08	0·92	5·16	0·91	<0·001
Race (white)	259	96·28	263	97·77	257	95·89	0·83
Gestational age							0·77
< 38 weeks	37	13·85	41	15·41	39	14·60	
38–40 weeks	110	41·19	111	41·72	101	37·82	
>40 weeks	120	44·94	114	42·85	127	47·56	
Birthweight (g)	3215	508·21	3243	519·2	3242	623·34	0·56
Birthweight							0·23
< 2500 g	24	8·98	23	8·64	30	11·23	
2500–3000 g	67	25·09	52	19·54	52	19·47	
3000–3500 g	106	39·7	116	43·6	94	35·2	
3500–4000 g	56	20·97	65	24·43	73	27·34	
> 4000 g	14	5·24	10	3·79	18	6·74	
Breastfeeding duration							<0·001
No breastfeeding	18	6·69	49	18·21	58	21·64	
< 6 months	77	28·62	85	31·59	90	33·58	
6–12 months	68	25·27	71	26·39	70	26·11	
> 12 months	106	39·40	64	23·79	50	18·65	
Child’s position among siblings							0·97
The oldest/singletons	106	39·40	99	36·80	94	35·07	
2nd/3rd or 2nd–3rd/4th	27	10·03	61	22·67	50	18·65	
The youngest or beyond the 4th	136	50·55	109	40·52	124	46·26	
*Z*-score of the BMI	−0·04	1·24	0·12	1·12	0·13	1·09	0·07
Nutritional status (g)							0·17
Low weight	46	17·1	36	13·38	31	11·56	
Normal weight	191	71	197	73·23	204	100·74	
Overweight/obesity	32	11·89	36	13·38	33	12·31	
Total energy intake (kcal)	2043	549	2092	486	2205	564	<0·001
Moderate-vigorous physical activity (h/d)	1·41	1·35	2·21	2·77	2·56	3·47	<0·001
Screen time (h/d)	0·97	1·06	0·97	0·66	1·21	0·86	<0·001

Among family factors, we found that children who reported high UPF consumption belonged to more numerous families (*P* < 0·001) and had slightly older mothers (*P* = 0·02). On the other hand, parental knowledge on children’s nutritional requirements (*P* < 0·001) and parental attitudes towards their child’s dietary habits (*P* < 0·001) were inversely associated with UPF consumption.

Regarding children’s characteristics, we observed that those who reported high UPF consumption were slightly older (*P* < 0·001), more physically active (*P* < 0·001), spent more time on screens (*P* < 0·001), but were less likely breastfed (*P* < 0·001). Marginally significant direct association was observed between UPF consumption and *Z*-score of the BMI (*P* = 0·07), but average *Z*-score of BMI was close to zero in all the categories and absolute differences were small.

The factors that independently predicted the consumption of UPF were family size, screen time, parental knowledge on children’s nutritional requirements, parental attitudes towards their child’s dietary habits and breastfeeding. In the multivariable-adjusted model (Table 2), we found that family size and screen time were directly associated with the consumption of UPF in childhood. However, higher nutritional knowledge, healthier dietary attitudes towards child’s dietary habits and longer breastfeeding predicted significantly lower UPF consumption in childhood. More specifically, children with three or more siblings consumed an average of 3·12 % (95 % CI (1·47, 4·78)) more energy from UPF than singletons or children with one sibling, and children in the second and third tertile of screen time consumed an average of 1·69 % (95 % CI (0·17, 3·21)) and 3·29 % (95 % CI (1·81, 4·77)) more energy from UPF, respectively, than their peers in the first tertile. On the other hand, children whose parents had medium or high nutritional knowledge consumed significantly lower energy form UPF than those whose parents had low knowledge (3·12 % (95 % CI (1·71, 4·88) and 5·95 % (95 % CI (3·84, 8·05) respectively). Along with this, parents with healthy dietary attitudes towards their child’s dietary habits had children who consume an average of 2·85 % (95 % CI (0·11, 5·59)) less energy from UPF than those with parents with unhealthy attitudes. Compared with children who did not breastfeed, those who breastfeed for 6–12 months and ≥ 12 months reported consuming significantly less energy from UPF (2·44 % (95 % CI (0·51, 4·38) and 4·98 % (95 % CI (2·94, 7·02)) respectively). All these factors together explained approximately 16 % of the variability in the consumption of UPF by children in this sample (coefficient of determination *R* ^2^ = 0·16)

**Table 2:** Adjusted differences* and 95 % CI in the percentage of TEI (% kcal/d) from ultra-processed foods associated with individual and family factors

	Adjusted difference (% kcal/d)		95 % CI	*P* value
Screen time (h/d)				
T1	0	Ref.		
T2	1·69		0·17, 3·21	0·02
T3	3·29		1·81, 4·77	<0·001
Number of children				
1–2 children	0	Ref.		
3 children	1·04		−0·43, 2·52	0·16
4 or more children	3·12		1·47, 4·78	<0·001
Attitudes towards child’s dietary habits				
Unhealthy	0	Ref.		
Average	−0·10		−2·93, 2·72	0·94
Healthy	−2·85		−5·59, −0·11	0·04
Knowledge about child’s nutritional recommendations				
Low	0	Ref.		
Average	−3·29		−4·88, −1·71	<0·001
High	−5·95		−8·05, −3·84	<0·001
Breastfeeding duration				
No breastfeeding	0	Ref.		
<6 months	−1·92		−3·81, −0·04	0·07
6–12 months	−2·44		−4·38, −0·51	0·02
> 12 months	−4·98		−7·02, −2·94	<0·001

Ref., reference.

* All the estimates are adjusted for the rest of the variables.

**Fig. 1:**
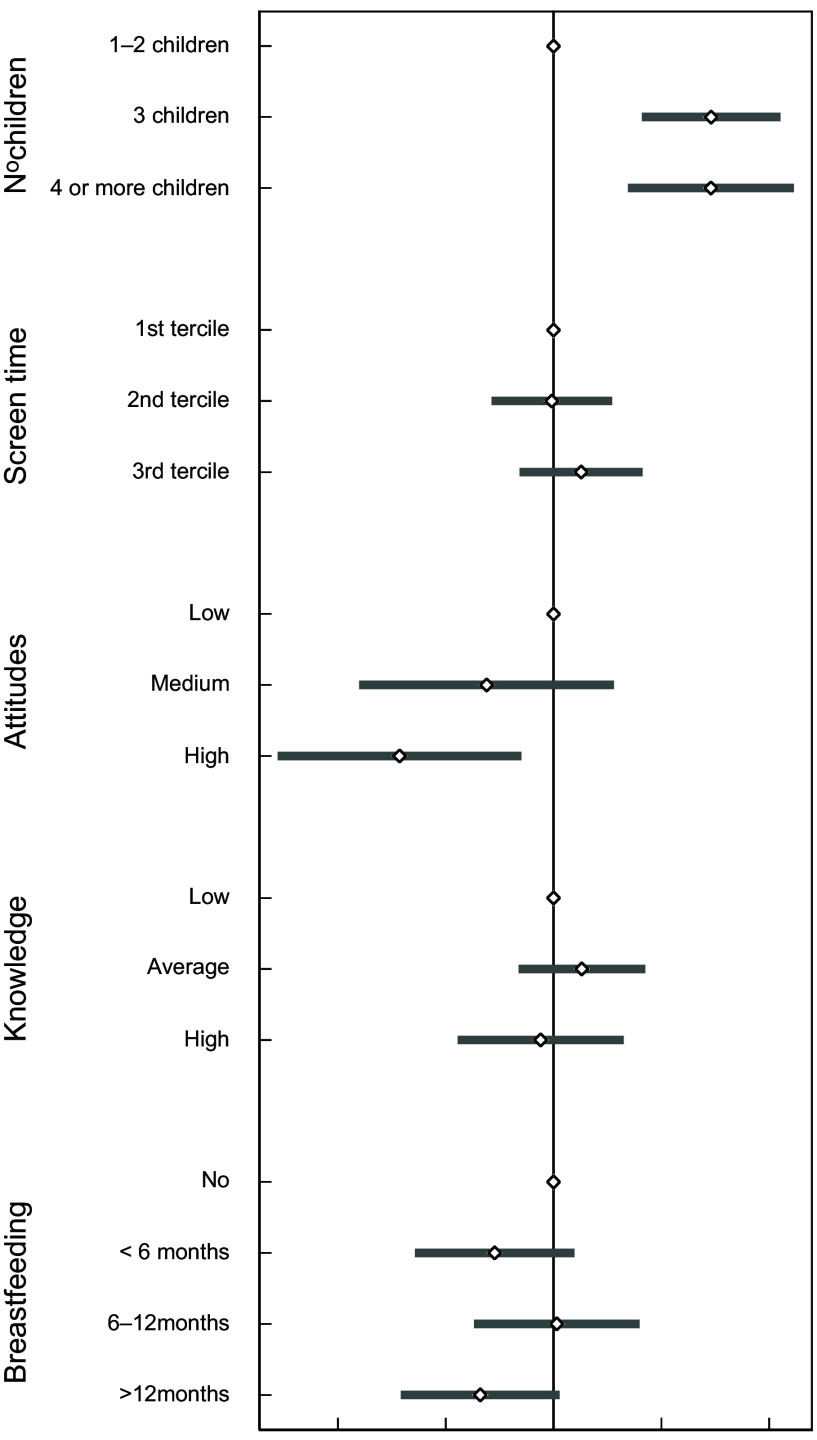
OR and 95 % CI for consuming more than 10 % of TEI as saturated fats associated with factors predicting the consumption of ultra-processed foods

**Fig. 2:**
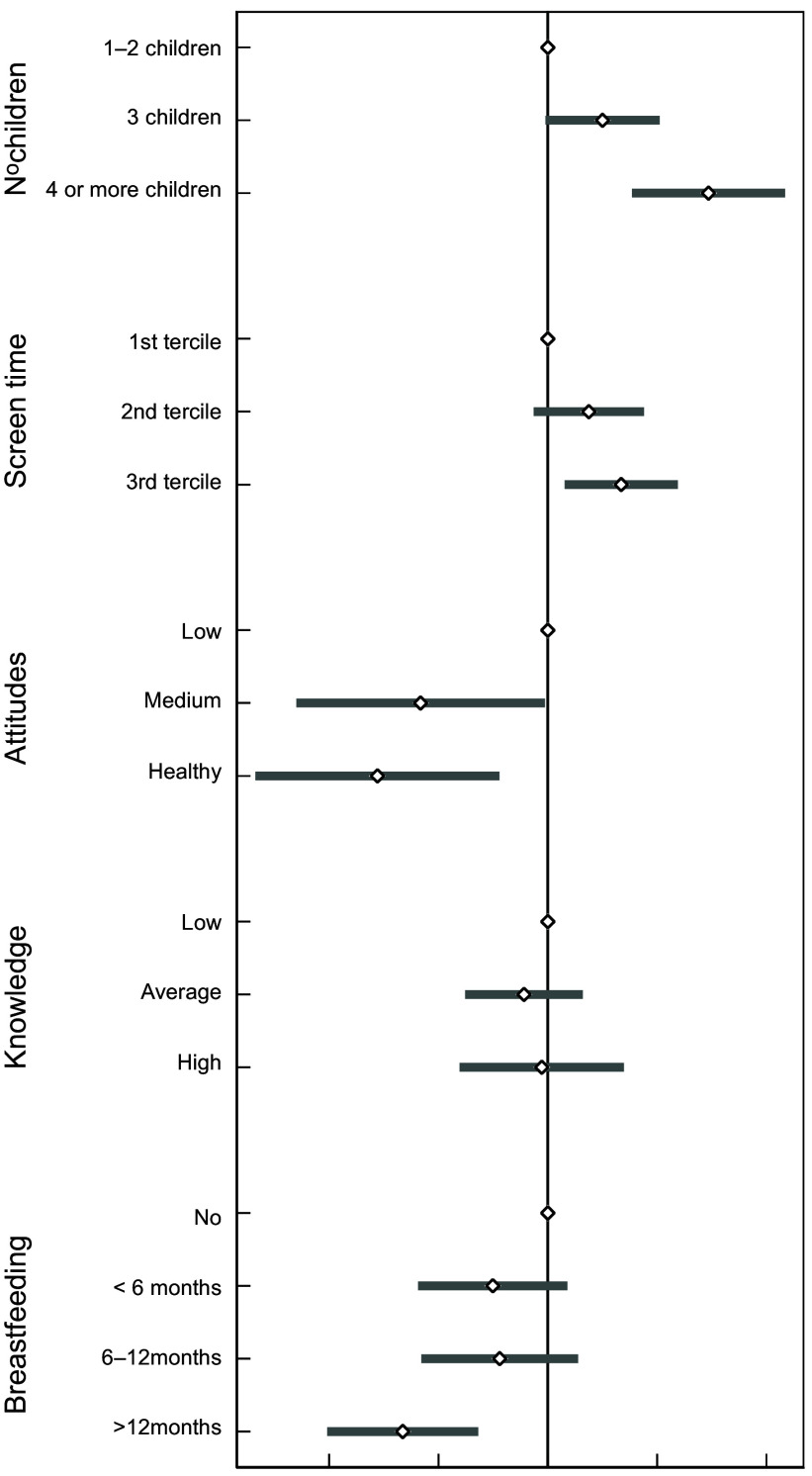
OR and 95 % CI for of consuming more than 10 % of TEI as free sugar associated with factors predicting the consumption of ultra-processed foods

In further analyses, we found that many of the factors previously found to be associated with UPF consumption were also consistently associated to higher odds of consuming more than 10 % of TEI from either saturated fats (Fig. 1) or free sugars (Fig. 2). Compared with singletons or children with one sibling, those with more than three siblings had an OR of 2·75 (95 % CI (1·76, 4·30)) for exceeding the daily fat intake recommendations and an OR of 2·76 (95 % CI (1·70, 4·50)) for exceeding the daily free sugar intake recommendations. Screen time emerged as an independent predictor of high free sugar but not of saturated fat intake. Children in the third tertile of screen time had an OR of 1·59 (95 % CI (1·11, 2·28)) for consuming more than 10 % of TEI from free sugars compared with children in the first tertile of screen time. After accounting for other factors, parental knowledge on children’s nutritional requirements was not an independent predictor for exceeding the WHO recommendations regarding saturated fats and free sugars intake. However, healthy parental attitudes towards their child’s dietary habits were associated with a relative reduction of 63 % (95 % CI (19, 84)) in the odds of consuming excessive saturated fats and of 67 % (95 % CI (27, 85)) in the odds of consuming excessive free sugar. Considering all the factors above, prolonged breastfeeding (≥12 months) was associated with lower odds of exceeding free sugar intake (OR: 0·39 (95 % CI (0·24, 0·64)), but no saturated fat intake.

## Discussion

In this study with 806 Spanish preschoolers, we identified several families and individual modifiable factors that predict UPF consumption in childhood. We found that children from numerous families and those with high screen time presented higher UPF consumption and were more likely to consume more than 10 % of TEI from free sugars. On the other hand, parental higher nutritional knowledge, healthier dietary attitudes towards their child’s eating habits and longer breastfeeding predicted lower consumption of UPF in childhood. Furthermore, parental dietary attitudes, but not nutritional knowledge reduced the odds of children exceeding the WHO recommendations regarding saturated fats and free sugars intake. Prolonged breastfeeding also reduced the odds of excessive intake of free sugars.

Many of the dietary and lifestyle habits of adulthood have been learned and fixed during childhood^(22)^. Since those factors significantly contribute to the development of obesity^(23)^ and other non-communicable diseases, the identification and early intervention aimed to modify those factors (at least those that are modifiable) becomes essential form a Public Health perspective.

Ecological and cross-sectional studies showed that UPF consumption was associated with a higher risk of overweight or obesity^(24,25)^ and other non-communicable chronic diseases^(26,27)^ among adolescents and adults, and although the evidence in children is scarce (maybe due to the induction time of some of those non-communicable chronic diseases), there is no evidence to suggest that the effects of UPF consumption in that population would be any different. However, there are very few studies aimed at identifying the catalysers of the determinants, yet it is essential to develop primary prevention strategies, which are the only ones that may reduce the incidence of a disease.

Two recent studies in Brazilian population showed that breastfeeding was associated with lower consumption of UPF^(28,29)^. Nevertheless, those studies had some limitations like the sample size^(30)^, the fact that the reference category was also breastfed^(30)^ and suboptimal control of confounding^(30,31)^. On the other hand, a large body of evidence showed that screen time and unhealthy dietary habits are highly pervasive and strongly associated in childhood^(31–33)^. Our study refines the existing literature because we identified several factors and calculated the independent effect of each one.

We previously published that parental attitudes towards child’s dietary habits were directly associated with child’s nutritional adequacy and diet quality^(30)^. Unlike the previous publication, in this study, we found that parental knowledge on children’s nutritional requirements was related to children’s consumption of UPF. Although it is possible that both parental knowledge and attitudes predict child’s habits, it seems logical that, after accounting for the other variable, attitudes would remain an independent predictor, but knowledge would not. Interestingly, in additional analyses, parental attitudes but not parental knowledge were associated with the odds of exceeding the WHO recommendations on free sugars and saturated fat intake. Further studies are needed to clarify to what extent the association observed in this study reflects a true association that we did not detect in the previous work due to lack of statistical power or the possibility that it is due to residual confounding.

To the best of our knowledge, the association between family size and diet quality has not been previously reported. In our opinion, the direct association observed between family size and UPF consumption may be explained, at least partially, by economic and time-related factors. Because UPF are cheaper and require less cooking time (many are sold as ready-to-heat or ready-to-eat products), it was not surprising to find that larger families relied more on these products. From a public health perspective, this finding highlights the strong need to increase the public subsidies to families for each additional children. In fact, the Spanish government provides the weakest economic support to families among all European countries^(34)^.

While UPF consumption is increasing worldwide, authorities have failed to set a clear threshold for healthy consumption. Meanwhile, higher consumption of UPF is leading to a higher intake of saturated fats and free sugars, which should represent less than 10 % of TEI according to the WHO recommendations. In this study, we found that several of the factors that predicted the consumption of UPF in childhood were also associated with the odds of exceeding those recommendations. Among the modifiable risk factors, parental attitudes towards child’s dietary habits emerged as the most important one since it was associated with lower odds of exceeding the intake of both saturated fat and free sugar intake. These findings highlight the importance of the family environment and the need for family interventions to improve children’s dietary habits^(23)^.

In univariate analyses, we found a direct association between physical activity and UPF consumption, but that association was not observed after adjusting for family size. The potential confounding effect of family size suggests that the more siblings a child has, the more time he/she spends in games and sports with moderate to vigorous physical demand. The analysis of participants’ dietary intake according to their physical activity level showed that more physically active children consumed a greater number of soft drinks and snacks, which are considered UPF according to the NOVA classification (data not shown). This suggests that there is a mistaken belief that soft drinks are healthy beverages for athletes, that an unhealthy habit can be compensated by a healthy one, or both^(35)^. It should also be kept in mind that exercise often leads to increased post-exercise snacking consumption ^(36)^.

Despite our findings, some limitations of our study must be acknowledged. First, due to the cross-sectional design, the possibility of a reverse causality bias of some of the factors identified as independent predictors of UPF consumption cannot be totally denied. More specifically, the relationship between screen time and UPF consumption may be bidirectional and therefore the magnitude of the association found in this study would be overestimated. These results should be reproduced in prospective studies before causality can be inferred. Second, we used self-reported information, which might lead to measurement errors. However, a validation study of self-reported and objectively measured weight and height showed correlations of 0·96 and 0·95, respectively, and concluded that information reported by participant’s parents was valid as to be used in epidemiological studies^(37)^. Although the FFQ was also previously validated, misclassification bias cannot be totally discarded. Nevertheless, there is no evidence to conclude that the validity of the reported information on lifestyle factors was associated with child’s consumption of UPF and, therefore, in case of an information bias, it would lead to a non-differential misclassification, which would, in any case, bias the estimate towards the null. The FFQ used in this study was not expected to collect information about food processing. To minimise a potential misclassification bias, food items classification was performed by two independent researchers and disagreements were solved by consensus. Four, participants in the SENDO project are mostly white children with highly educated parents from a developed European country. Although these factors may hamper the generalisability of our results, it has some benefits, such as higher validity of the self-reported information and the reduction of potential confounding by socio-economic variables^(38)^. Fifth, due to the observational design of the study, we must acknowledge the possibility of residual confounding for variables that were not collected such as socio-economic status. Lastly, although the questionnaires about parental knowledge on children’s nutritional requirements and that parental attitudes towards child’s dietary habits have not been validated yet, previous studies showed they are associated with diet quality in both paediatric and adult populations^(39–41)^.

Our study has several strengths, including the large sample size. The baseline questionnaire of the SENDO project is extensive, which allows the evaluation of several factors. To the best of our knowledge, this is the first study assessing the association of several modifiable risk factors and UPF consumption in childhood in a single multivariable model and, therefore, controlling for the potential confounding effect of each other. Lastly, we accounted for intra-cluster correlation between siblings in all the analyses, which is a common limitation of studies in paediatric populations.

In conclusions, we found that children from numerous families and those with high screen time consume more energy from UPF than those from small families and little screen time. Furthermore, higher parental knowledge on children’s nutritional requirements, healthier attitudes towards child’s dietary habits and prolonged breastfeeding are associated with lower consumption of UPF. Parental attitudes towards child’s dietary habits emerged as an independent predictor of compliance with WHO recommendations regarding saturated fats and free sugar intake. The consistency of the findings for that factor and the doses–response relationship suggest that the observed association is not explained by just residual confounding, but might reflect a true biological effect. These results highlight the importance of modifiable factors in the primary prevention of non-communicable diseases and suggest that interventions aimed at promoting healthy dietary habits in childhood should focus on the whole family and not only on children.

## Supplementary material

For supplementary material accompanying this paper visit https://doi.org/10.1017/S136898002200132X. click here to view supplementary material
