# Supplementary material for: Individual and family predictors of ultra-processed food consumption in Spanish children: The SENDO project
Source: Public Health Nutr. 2022 May 27;26(2):437–45. doi: 10.1017/S136898002200132X (PMC13076079; doi:10.1017/S136898002200132X)
Supplement: Supplementary file 1 [file S136898002200132Xsup001.docx]

**Supplementary table 1: Classification on foods in the SENDO food frequency questionnaire according to the** **extent and purpose of processing by NOVA.**

| **Unprocessed or minimally processed foods** | **Processed culinary ingredents** | **Processed foods** | **Ultra-processed food and drink products** |
| --- | --- | --- | --- |
| **Apple, asparagus, eggplant, avocado, banana,** , beans, cabbage, carrot, chard, cherry, chicken, clam, curd, eggs, fig, fish, fruit juice, fruit smoothie, garbanzo beans, grapes, kiwi fruit, lamb, leek, lentils, lettuce, mango, meatball, melon, milk (skimmed or whole), nuts, octopus, onion, orange, pasta, peas, peach, pear, pepper, pineapple, plum, pork, potatoes, seafood, pumpkin, rabbit meat, rice, strawberry, string beans, tangerine, tomato, veal, viscera,watermelon | Sunflower oil, olive oil, sugar, butter, cream, salt. | Olives, compote of fruit, cured ham, canned fish, jam, baguette, wholemeal bread, white cheese, cured cheese,bacon | Bakery products, blood sausage, bonbon, breakfast cereals, cake, candies, carbonated beverages, cereal bar, chocolate bar, chocolate powder, cookies, crab sticks, cream cheese, cream chocolate, croquet*, cruller or ‘churro’*, custard*, dry soup, fish sticks, gelatine, ham, hamburger, ice cream, industrialised juices (sugar-sweetened juices), industrialised sliced cheese, industrialised sliced bread, ketchup, lasagne*, margarine, mayonnaise*, muffin*, nougat, nugget, pâté, petit suisse, pie*, pizza*, popcorn*, salami, sausage, pepperoni, snacks, soda, soft drinks, sweetened beverages, sweetened fermented milk, sweetened yogurt (skimmed or whole) |

*There are foods that could have different ratings depending on the way they are prepared: homemade or industrialised. In these cases, we chose to classify them as ultra-processed foods because most traditional foods have been replaced by industrial food products in supermarkets

**Supplementary Table 2: Paternal attitudes towards child’s dietary habits.**

| **I try my child to…** | **NO** | **YES** |
| --- | --- | --- |
| Eat more fruit? | 0 | 1 |
| Eat more fiber? | 0 | 1 |
| Eat more vegetables? | 0 | 1 |
| Eat more fish? | 0 | 1 |
| Avoid butter consumption? | 0 | 1 |
| Reduce fat intake? | 0 | 1 |
| Reduce meat consumption? | 0 | 1 |
| Reduce eating sweets and pastries? | 0 | 1 |
| Total (min/max) | 0 | 8 |

**Supplementary Table 3: Parental knowledge about nutritional recommendations for children.**


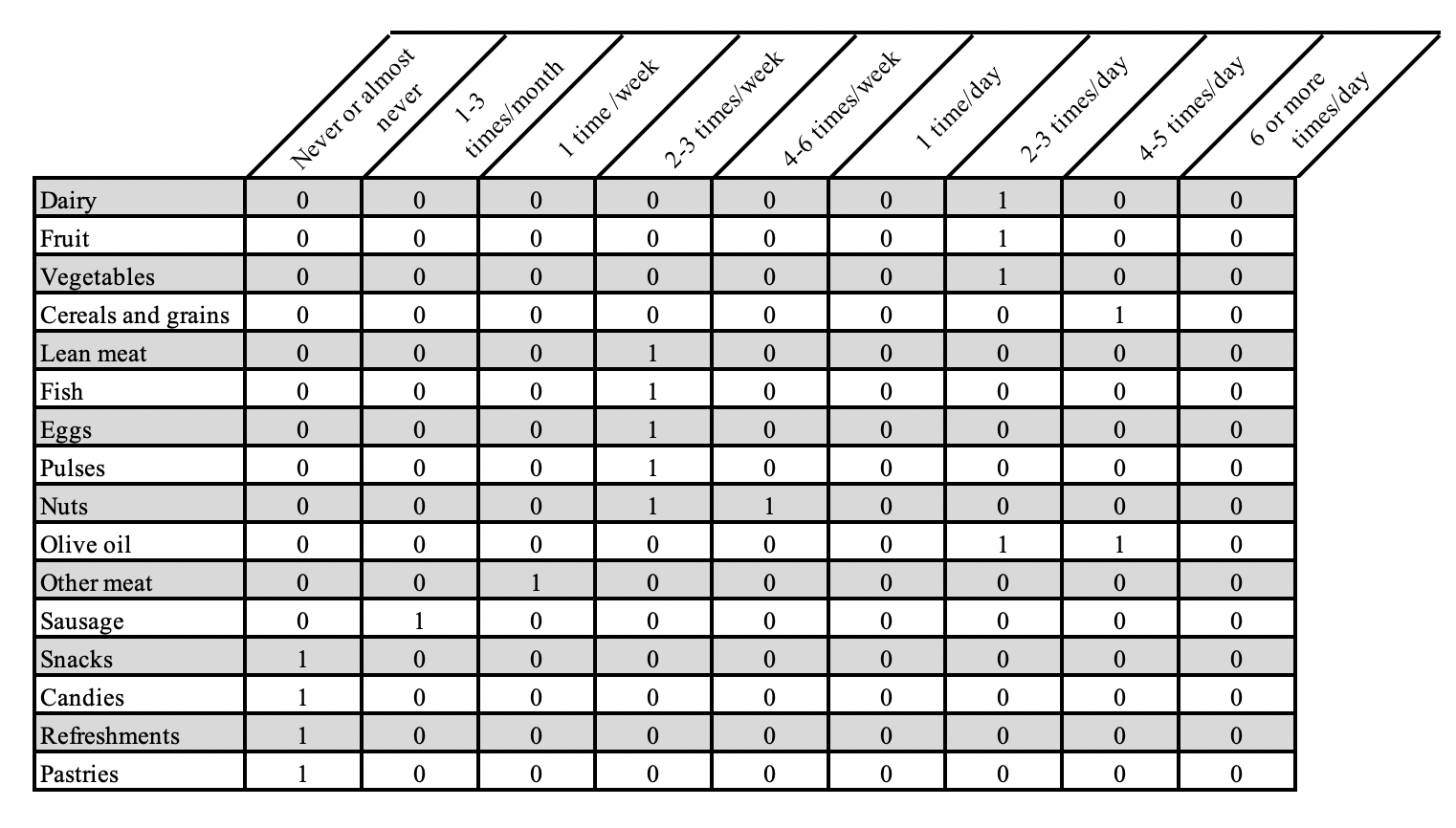
How often do you think your child should consume these foods to follow a healthy diet?
